# Multi-Contrast Multi-Atlas Parcellation of Diffusion Tensor Imaging of the Human Brain

**DOI:** 10.1371/journal.pone.0096985

**Published:** 2014-05-08

**Authors:** Xiaoying Tang, Shoko Yoshida, John Hsu, Thierry A. G. M. Huisman, Andreia V. Faria, Kenichi Oishi, Kwame Kutten, Andrea Poretti, Yue Li, Michael I. Miller, Susumu Mori

**Affiliations:** 1 Center for Imaging Science, Johns Hopkins University, Baltimore, Maryland, United States of America; 2 Department of Electrical and Computer Engineering, Johns Hopkins University, Baltimore, Maryland, United States of America; 3 Russell H. Morgan Department of Radiology and Radiological Science, Johns Hopkins University School of Medicine, Baltimore, Maryland, United States of America; 4 Department of Biomedical Engineering, Johns Hopkins University School of Medicine, Baltimore, Maryland, United States of America; 5 F.M. Kirby Research Center for Functional Brain Imaging, Kennedy Krieger Institute, Baltimore, Maryland, United States of America; Beijing Normal University, China

## Abstract

In this paper, we propose a novel method for parcellating the human brain into 193 anatomical structures based on diffusion tensor images (DTIs). This was accomplished in the setting of multi-contrast diffeomorphic likelihood fusion using multiple DTI atlases. DTI images are modeled as high dimensional fields, with each voxel exhibiting a vector valued feature comprising of mean diffusivity (MD), fractional anisotropy (FA), and fiber angle. For each structure, the probability distribution of each element in the feature vector is modeled as a mixture of Gaussians, the parameters of which are estimated from the labeled atlases. The structure-specific feature vector is then used to parcellate the test image. For each atlas, a likelihood is iteratively computed based on the structure-specific vector feature. The likelihoods from multiple atlases are then fused. The updating and fusing of the likelihoods is achieved based on the expectation-maximization (EM) algorithm for maximum a posteriori (MAP) estimation problems. We first demonstrate the performance of the algorithm by examining the parcellation accuracy of 18 structures from 25 subjects with a varying degree of structural abnormality. Dice values ranging 0.8–0.9 were obtained. In addition, strong correlation was found between the volume size of the automated and the manual parcellation. Then, we present scan-rescan reproducibility based on another dataset of 16 DTI images – an average of 3.73%, 1.91%, and 1.79% for volume, mean FA, and mean MD respectively. Finally, the range of anatomical variability in the normal population was quantified for each structure.

## Introduction

For quantitative analysis of the human brain anatomy, defining structures or regions of interest (ROIs) is one of the first essential steps. There are many types of automated or manual approaches that have been proposed to define ROIs in the brain, based on locations and contrasts of the structures. These methods often require a *priori* knowledge as a form of atlas. For manual ROI drawing, an atlas could be a simple pictorial representation of a structure of interest, which guides operators to define the boundary. The manual delineation, while often used as a gold standard, is a time-consuming approach. Various types of automated parcellation tools have been proposed, which try to define the boundary of anatomical structures based upon image contrasts [1]–[17]. Some of the advanced tools incorporate a *priori* knowledge about the location of the target structures as a form of probabilistic atlas [18]–[26]. This location constraint prevents the contrast-based boundary definition from leaking into unlikely regions.

To use the probabilistic location information in the atlas, the atlas has to be registered, or warped, to each subject image, in which voxel-to-voxel correspondence is established between the two coordinate systems where the atlas image and the subject image are defined. The concept of brain mapping also leads to an alternative approach for automated structural parcellation, which is called atlas-based parcellation [3], [7], [11], [27]–[35]. Namely, if the voxel-to-voxel mapping is perfectly accurate, any arbitrary structures can be defined only once in the atlas and such anatomical definitions can be transferred to the images being mapped to. There are no constraints in the number of structures or the way the structures are defined in the atlas. In this sense, we can assume that the whole-brain voxel mapping inherently contains parcellation tools for potentially all definable structures inside the brain.

The atlas-based parcellation, however, is accurate only if the voxel-to-voxel brain mapping correctly defines corresponding voxels between the two images, which is not always guaranteed. The accuracy level is influenced by various sources of differences between the atlas and the subject brains; these could be morphological (atrophy, hypertrophy, malformation, etc.) or contrast (biological such as signal hyperintensity or hypointensity or procedural such as imaging parameters).

To reduce the impact of erroneous mapping of voxels, and consequent mis-parcellation of target structures, multi-atlas approaches have been postulated. Suppose that the hippocampus is defined in 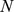
different atlases and each atlas is warped to a to-be-parcellated subject image, then the 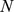
definitions (labels) of the hippocampus are casted to the subject space, which can be fused (a.k.a “label fusion”) based upon pre-defined algorithms such as those proposed by [7], [36]–[42]. If the mis-registration of each atlas causes random errors, the errors should be reduced by integrating 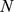
definitions. It has been shown that simple label fusion techniques based on majority voting yield robust parcellations [7], [41], [43]. More recently, weighted majority voting strategies, by incorporating intensity information, demonstrated significant improvement in the parcellation accuracy. A variety of weighting approaches, based on intensity similarity metrics, have been proposed – global [36], local [44], [45], semi-local [45], [46], and non-local [47]. In addition to voting, a statistical fusion technique (i.e. Simultaneous Truth and Performance Level Estimation, STAPLE [42]) and a collection of its variants [48]–[50] have been proposed, in which a stochastic model of rater behavior has been incorporated in the estimation process. Compared with voting techniques, the main limitation of statistical fusion strategies is that the decision rule is independent of the image intensity while the major advantage is its underlying elegant mathematical theory. Initial attempts to incorporate the intensity information into the STAPLE framework rely on a priori similarity measures [51], [52] or estimating the voxelwise correspondence between the registered rater and the subject using intensity information [49].

We have recently introduced the diffeomorphic likelihood fusion algorithm (DLFA) as an approach to integrate anatomical information from multiple T1-weighted atlases and fuse their anatomical features [53], [54]. Unlike previous label fusion algorithms, DLFA does not fuse a set of binary label maps obtained from the atlas-to-subject propagations. DLFA poses the parcellation problem in the framework of maximum a posteriori (MAP) estimation, estimating the maximizing parcellation labels given the observable image intensity, similar to the idea proposed in [6]. The MAP estimation is handled within the class of generative models by representing the observable imagery as a conditionally Gaussian mixture random field, conditioned on the random atlas-label pair and the diffeomorphic change of coordinates for each label. The atlas-label pair and their diffeomorphic correspondences are unknown and viewed as latent variables. Locality is introduced into the global representations of the deformable templates by allowing different atlas-label pairs to be used to interpret different voxels or different structures, under the assumption that the local optimal diffeomorphism varies from label to label for a given atlas. The MAP estimation is solved by iterating between fixing the local optimal diffeomorphisms and obtaining the maximizing parcellation labels, and then locally optimizing the local diffeomorphisms for the fixed parcellation, in an EM fashion [55]. The atlas-dependent structure-specific local diffeomorphisms are estimated in the E-step in the EM algorithm.

The purpose of this paper is to extend the DLFA to diffusion tensor imaging by incorporating multiple-contrast information. It arises naturally to extend a single contrast image such as T1-weighted images to multi-contrast images (e.g. eigenvalues and eigenvectors of DTI) by assuming conditional independence in computing 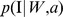, where 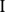
denotes the measurable image, 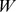 denotes a given parcellation label, and 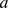 the randomly selected atlas-label pair. Previously, vector-to-vector or tensor-to-tensor registration algorithms have also been introduced [56]–[59], which was further extended to multi-channel image registration, in which multiple-contrast information, such as FA, diffusivity, and fiber orientation, is used simultaneously to drive the registration algorithm [60]. These ideas could improve the registration accuracy between each atlas and the testing subject, but the incorporation of the multiple-contrast information in the multi-atlas likelihood fusion process has not been introduced so far.

In this paper, we introduce a framework to incorporate the multi-contrast intensity information generated in DTI into the multi-atlas DLFA framework and apply it to whole brain parcellations into 159 structures. In the T1 case, the distribution of the intensity in each structure is modeled as a single Gaussian. In the DTI case, we use five intensity elements ([FA, MD, and fiber angle (a unit vector)]) with the intensity distribution of each, in every single label, being modeled as a Gaussian Mixture Model (GMM), the parameters of which are computed using maximum-likelihood estimation. In this study, we examine the parcellation accuracy of the method on 25 patient data with a varying degree of pathology. We also present the scan-rescan reproducibility of the method on another dataset of 16 healthy subjects which were scanned twice. In addition, the ranges of anatomical variability of all the structures in the 16 subjects were characterized.

## Methods and Materials

### Patient populations

All subjects used as atlases and the first testing dataset were obtained from the existing clinical database of pediatric brain MRI, and were older than 24 months of age. DTIs from sixteen subjects (Female = 7, Male = 9, age = 7.67+/−4.12) were used to create the multiple atlases (Table 1). Among these sixteen subjects, ten subjects were diagnosed as normal. In order to cover the wide range of anatomical phenotypes in the multiple atlases, 6 cases with different types of anatomical abnormalities were also included in the atlas set as shown in Table 1.

**Table 1:** Anatomical changes in the sixteen subjects used as the multiple atlases.

Atlas ID	Radiological findings	Radiological diagnosis
1	White matter T2 hyperintensity involving the bilateral periventricular and deep white matter with restricted diffusion spots	Drug-induced leukoencephalopathy
2	T2-hyperintense lesions in periventricular and subcortical white matter	Multiple sclerosis
3	Multiple encephalomalacia/gliosis change related to sequela from prior ischemic events	Moyamoya-disease
4	Diffuse CSF space dilatation	Associated finding with achondroplasia
5	Multiple T2-hyperintense lesions in white matter and gray matter	Neurofibromatosis type1
6	Mild ventricular dilatation with irregular shape and volume loss of periventricular white matter with posterior dominant	Periventricular leukomalacia
7∼16	No abnormal finding	Diagnosed as normal

The accuracy of the parcellation obtained from multi-atlas DLFA was tested using 25 patients (Female = 10, Male = 15, age = 7.88+/−4.80). As tabulated in Table 2, 10 subjects (Test #1–#10) presented a normal MR anatomy and the other 15 subjects presented a variety of anatomical abnormalities; seven (Test #11–#17) were evaluated as mild to moderate anatomical change and eight (Test #18–#25) as severe abnormality based on a pediatric neuroradiologist's (S.Y.) visual evaluation.

**Table 2:** Anatomical changes in 25 subjects for testing the multiple atlases application.

Test Subject ID	Radiological findings	Radiological diagnosis
1∼10	No abnormal finding	Diagnosed as normal
11	Mild deep white matter T2-hyperintense change and ventricle enlargement	Adrenoleukodystrophy
12	Right hemiatrophy, ventricle dilatation and mild T2-hyperintense change in deep gray matter	Chronic ischemic insult
13	Diffuse CSF space and ventricle dilatation	Associated finding with achondroplasia
14	Mild ventricle dilatation	Associated finding with achondroplasia
15	T2-hyperintense lesions in periventricular and subcortical white matter and mild ventricle enlargement	Multiple sclerosis
16	Porencephalic left ventricle dilatation and volume loss of left corticospinal tract	Prenatal hemorrhagic insult
17	Asymmetrical ventricle dilatation (right>left)	Associated finding with achondroplasia
18	Ventricle enlargement with multiple T2-hyperintense lesions in white matter	Multiple sclerosis
19	Ventriculomegaly associated periventricular volume loss (right>left) and T2-hyperintense change	Perinatal hypoischemic injury
20	Lateral ventricular enlargement with periventricular white matter volume loss and T2-hyperintense change	Periventricular leukomalacia
21	Right ventricle enlargement associated with periventricular white matter volume loss	Prenatal intraventricular hemorrhage
22	Diffuse parenchymal volume loss, CSF space dilatation and multiple ischemic lesions	Congenital metabolic disease
23	Ventriculomegaly and thinning of corpus callosm	Ventriculomegaly
24	Left hemiatrophy	Sturge-Weber syndrome
25	Left parenchyamal volume loss, gliosis and lateral ventricle enlargement	Perinatal stroke

For the scan-rescan reproducibility test, sixteen healthy volunteers with no history of neurological conditions (8 M/8 F, 22–61 years old, mean: 31 years old) participated in this study. This is the same data used by [61], where details of the protocol can be found.

### Ethics Statement

This study was approved by the Johns Hopkins Medicine Institutional Review Board (JHM-IRB). All subjects provided written, informed consent for participation in accordance with the oversight of the JHM-IRB.

### MRI scans

For the first dataset, MR imaging was performed using a 1.5T scanner (Avanto; Siemens, Erlagen, Germany). All patients underwent routine clinical multiplanar T1, T2, and FLAIR pulse sequences, including DTI. The DTI was obtained using a single-shot EPI with parallel acquisition. Diffusion weighting was performed along 21 independent axes with b = 1000 s/mm2, and repeated twice to enhance the SNR (TE = 84 ms, TR = 7700 ms). DTI was scanned in the axial orientation with an imaging matrix of 96×96 (to 192×192 with zero-filled interpolation), FOV 240×240, and slice thickness 2.5 mm.

For the second dataset, subjects were scanned twice using a 3T MR scanner (Achieva, Philips Healthcare, Best, The Netherlands). The DTI dataset was acquired using a multi-slice, single-shot, echo-planar imaging (EPI), spin-echo sequence (TR/TE = 6281/67 ms, SENSE factor = 2.5). Sixty-five transverse slices were acquired parallel to the line connecting the anterior commissure (AC) to the posterior commissure (PC) with no slice gap and 2.2 mm nominal isotropic resolution (FOV = 212×212, data matrix = 96×96, reconstructed to 256×256).

### DTI processing

All DTI datasets were processed offline using DTIStudio software (H. Jiang and S. Mori, Johns Hopkins University, Kennedy Krieger Institute, lbam.med.jhmi.edu or www.MriStudio.org) [62]. The raw diffusion-weighted images were first co-registered to one of the b0 images with a 12-parameter affine transformation using Automated Image Registration (AIR) [63]. The six elements of the diffusion tensor, the fractional anisotropy (FA), and the mean diffusivity (MD) were calculated.

### Initial creation of multiple atlases

For the sixteen subjects that were selected to be the multiple atlases, the images were first normalized to MNI coordinates with a nine-parameter affine transformation. The initial parcellation of the brain into 159 structures was performed using the atlas-based automated image parcellation pipeline as described in our previous publication [60]. We used our single-subject Eve atlas [64] and the accompanied brain parcellation map with 159 structural definitions as the template, which was warped to the 16 subjects using the three-contrast large deformation diffeomorphic metric mapping (LDDMM) [64]–[66]. The three contrasts included FA, MD, and the manually-delineated lateral ventricles. In a previous study, we tested the accuracy of this automated structural parcellation approach in cerebral palsy patients and excellent accuracy was reported [65]. In this study, we included patients with more severe abnormalities. If gross parcellation errors occurred, they were manually corrected to establish the multiple atlases with accurate structural definitions. The non-linear image transformation and the atlas-based parcellation were performed using DiffeoMap and RoiEditor (http://www.MriStudio.org, Kennedy Krieger Institute and Johns Hopkins University, X. Li, H. Jiang, and S. Mori). The atlas data are available at http://lbam.med.jhu.edu.

### Multi-contrast likelihood-fusion

Let 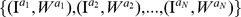
denote 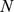 DTI atlas-label pairs, where 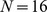 in this study. Instead of using single-valued (T1-weighted) images, we use vector-valued images for both the atlases and the test subjects. For each atlas-label pair 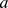, 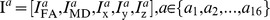, where 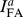
denotes the gray-scale FA image of the atlas-label pair, 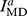
denotes the gray-scale MD image of the atlas-label pair, and 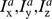 denote the absolute values of the three elements of the primary eigenvector. In this sense, the image intensity at each voxel is a 5-element vector 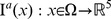, with 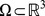 being a finite grid where the images are defined. For the label image 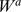 in each atlas-label pair, we define it as a function from the image domain 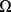 to a subset of the non-negative integers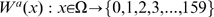, where 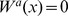 for voxel 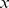 belonging to the unlabeled background, and 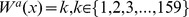 for voxel 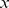 labeled as the 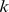-th structure such as the left caudate, the right putamen, and so on. Correspondingly, we denote the to-be-parcellated test subject as 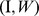, where 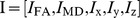 and 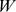 is the label image we aim to obtain.

For multi-contrast, multi-atlas parcellation, the goal is to estimate the label map 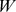 associated with the image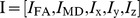 of the test subject, for which we solve via the Maximum a Posteriori (MAP) estimation
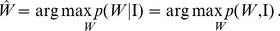
(1)To achieve this goal, we use the EM algorithm by introducing the latent variable 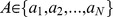
that designates the random atlas-label pair. The *Q*-function in the EM algorithm computes the log-likelihood of the complete data 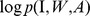 given the incomplete data — the to-be-parcellated measured image 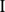 and the previous parcellation label 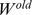,
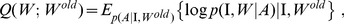
(2)where
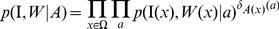
(3)with 
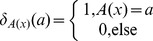
 indicating that 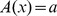 is used to interpret the voxel 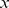
in the test image. Denoting the conditional probability of the atlas-label selector as 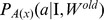, the Q-function reduces to:
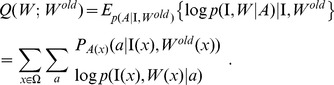
(4)The sequence of iterates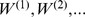, associated to the alternating maximization defined by the iteration
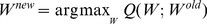
(5)is monotonic in the incomplete data likelihood with atlas selector 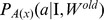, the proof of which can be found in [53].

The algorithm can be summarized as:

Step1: Initialize the diffeomorphism for each voxel 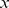 to be identical everywhere, as: 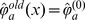. Initialize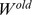.

Step2: Compute the approximated atlas-label selector as:
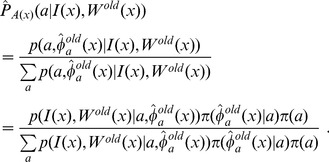
(6)Step3: Obtain a new parcellation image for the test image via 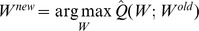, where 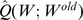
is computed as:
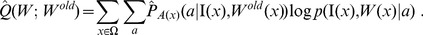
(7)Step4: Recalculate the diffeomorphisms of the atlases onto the parcellation labels via: 
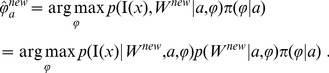
(8)Step5: Update the parcellation 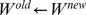 and the optimal diffeomorhiphisms 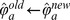, go to Step 2.

Stop the iteration if either 
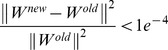
 or the number of total iterations is bigger than 30.

#### Remarks

1. To initialize the optimal diffeomorphism 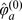 in Step 1 that is associated to the atlas-label pair 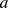, we used the optimal diffeomorphism obtained from a two-channel LDDMM image mapping with one channel being the FA images and the other the MD images, which has been validated in registering DTI images [60]. Given the pair of the target 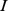
and an atlas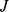, we compute a diffeomorphic deformation 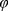
between the two vector valued images 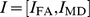
and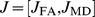 such that 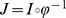 or 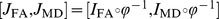. The diffeomorphism is assumed to be generated as the end point, 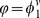, of the flow of the smooth time-dependent vector field, 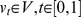, via the ordinary differential equation 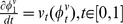, where 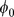
is the identity transformation. The optimal diffeomorphic deformation is generated by integrating the vector field, which is found to minimize the energy:
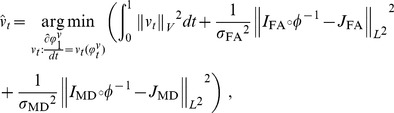
(9)where the parameters 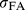
and 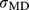 control the weighting of the two contrast-matching terms of smoothness regularization terms. In this study, we set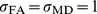. To ensure that the solution of Eq. (9) lies in the space of diffeomorphisms, the set of time-indexed vector fields 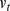 must be sufficiently spatially smooth, requiring 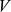
to be a reproducing kernel Hilbert space. For computational purposes, we use an operator-induced norm on 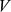
such that 
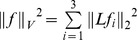
 and 
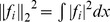
 with the differential operator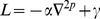, where 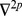
is the Laplacian operator with power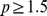. In this study, we use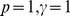, and 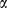
is selected according to the cascading method described in [60], [67] as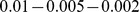.

For the initialization of the parcellation label in Step1, there are multiple choices. In our case, we use the propagation of the labels of atlas-label pair 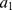
under the optimal global diffeomorphism 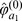---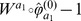.

2. To incorporate the local optimized diffeomorphism 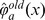 in the calculation of Eq. (6), we use mode approximation via
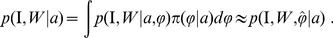
(10)The optimized diffeomorphism in Eq. (10) is obtained as the mode, as computed in Eq. (8).

3. In calculating the terms in Eq. (6), we assume that the prior distribution on the atlas-label pair
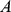
is uniform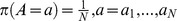. Via Bayes' rule, we have: 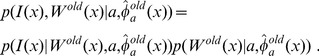
(11)To calculate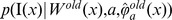, we define a hierarchical model between the image 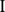
and the underlying diffeomorphic change in coordinates of the atlas 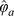, so that 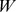
splits 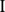
and 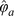. Conditioned on 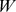, the joint measurement, 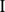 and 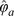, is independent, giving rise to:
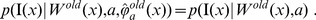
(12)Therefore, we have:
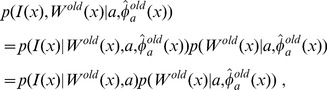
(13)where 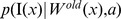
is computed as 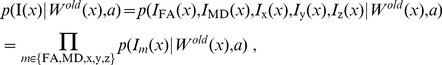
(14)with 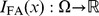 indicating the FA value at voxel 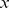 in the target. In calculating each single term in Eq. (14) such as 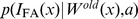, we model it as the probability density function of a Gaussian Mixture Model (GMM), the parameters of which are computed from the atlas-label pair. To be specific, for atlas-label pair 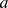, we model
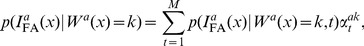
(15)where 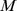
denotes the total number of Gaussians in the mixture model, 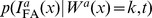 represents the probability density function of a single Gaussian
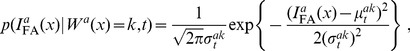
(16)and 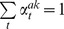 are the mixing coefficients for different Gaussians. For the parameters of the mixture of Gaussians associated to a specific label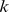, 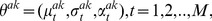
(17)we employ the EM algorithm to derive the maximum-likelihood estimators. The term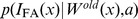 in Eq. (14) is computed according to
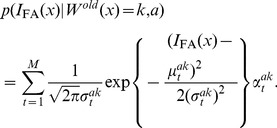
(18)For any given structure, the total numbers of Gaussians, 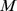, for the mixtures are pre-defined. We set 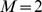 for the structures smaller than 1000 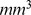 and 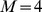 for those larger than 1000 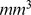. These parameters in the GMM were empirically determined. The GMM is used to quantitatively characterize the characteristic shape of the histogram of the intensity distribution of each contrast in each structure.

4. To compute the Q-function as described in Eq. (7), according to Bayes' rule, we have 
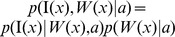
. The term 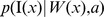
is computed as demonstrated in Eqs. (14) - (18), and 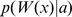 is approximated via 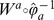 under trilinear interpolation.

5. Given our splitting assumption in Eq. (12), Eq. (8) is equivalent to 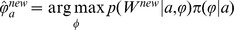. Considering computational efficiency, we use measures of the distance between the parcellation of the target and the diffeomorphically deformed results of the atlas parcellations, analogous to LDDMM for image matching and surface matching. To be specific, we use the Dice overlap measurement between 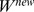
and 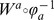 to approximate the term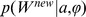. The optimal local diffeomorphism is assumed to come from a composition of the optimal global diffeomorphism and an optimal local 12-parameter affine transformation. Namely, 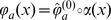, where 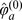 is the optimal global diffeomorphism computed in Step 1 and 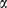
is the optimal local 12-parameter affine transformation that maximizes 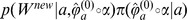, where 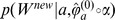
is quantified as the Dice overlap between 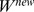
and 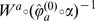. Note that the optimal local affine transformation is obtained on a structure-by-structure basis. Therefore, for a single atlas-label pair 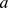, the optimized local diffeomorphisms 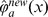 should be identical for voxels in the same structure. Given atlas-label pair 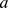, the prior distribution of the transformation 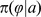 is estimated as the multiplication of two terms 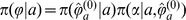, where 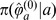
is estimated by the one over the metric distance [68] in diffeomorphism space given by the exponential of the geodesic length, computed from the two-channel LDDMM mapping. The prior on the 12-parameter affine transformation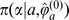 is modeled as a multivariate Gaussian, 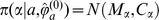, similar to the strategy adopted in [69]. In our approach, we use 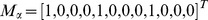
and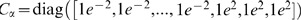. We assume that all the parameters are mutually independent, and thus the covariance matrix is diagonal. Since the first 9 parameters in 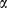
represent the affine matrix, their variances should be small, for which we assign 0.01. The last 3 parameters represent the translation in the 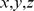
directions, and therefore their variances should be big, for which we use 100.

Since the optimization of the local diffeomorphisms is based on the overlap between the parcellation 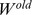
of the target and the diffeomorphically deformed results of the atlas parcellations and the optimized diffeomorphisms 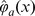 are identical for voxels in the same structure, the term 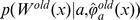
in Eq. (11) is approximated as being proportional to the overlap distance between 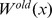 and 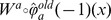. Again, for atlas-label pair 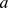, this quantity is identical for voxels in the same structure.

To sum up, the MAP estimation problem is solved in an EM approach. We iterate between fixing the local optimal diffeomorphism for each label in each atlas-label pair and obtaining the maximizing parcellation of the target, and then locally optimizing the diffeomorphisms associated to each label in each atlas-label pair given the fixed parcellation.

### Image quantification

After the brain had been parcellated to the 159 structures, the peripheral ROIs were further decomposed to the CSF, cortex, and peripheral white mater using MD (threshold value = 0.0015 to separate the CSF and the tissue) and FA (threshold value = 0.2 to separate the cortex and the white matter) (Faria et al., 2010; Oishi et al., 2009). The CSF regions were excluded from the analysis. There were 50 peripheral ROIs and thus the final number of ROIs was 193. The volumes of these ROIs were obtained by counting the number of voxels. ROI-specific FA and MD values were measured by averaging the values of all voxels within the ROIs. The parcellation criteria used in this paper followed our previous publications [70], [71], in which the cortex and the white matter definitions followed ICBM-LPBA40 [24] and probabilistic white matter atlas [71], respectively.

### Manual delineation for accuracy measurements

Eighteen structures (sixteen white matter structures and two deep gray matter structures) were manually delineated on the pre-selected 2D slices of fourteen subjects from three groups – four from the normal group, five from the mild abnormal group, and five from the severe abnormal group. The manual delineation was performed by incorporating information from MD, FA, and color-coded eigenvector maps. To investigate the intra- and inter-rater variability of the manual delineations, two raters (X.T. and J.H.) performed the manual delineations twice with more than 3-week intervals. To quantitatively evaluate the parcellation accuracy of our algorithm, we used four measurements:

1. Dice overlap coefficients

We calculated the Dice overlap coefficients between the manually delineated 2D ROI and the corresponding ROI in the automated parcellations. The Dice overlap coefficient is calculated as: 
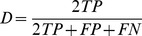
, where 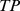 is the area of the region that belongs to both the automated ROI and the manual ROI, 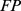 is the area of the region that belongs to the automated ROI but not the manual, and 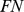 is the area of the region that belongs to the manual ROI but not the automated.

2. The correlation between the size of the manually delineated ROI and that of the automated ROI.

3. The correlation between the mean FA value of the manual ROI and the mean FA of the automated ROI.

4. The correlation between the mean MD value of the manual ROI and the mean MD of the automated ROI.

To evaluate the improvement in parcellation accuracy given by the multi-contrast approach, we compared the parcellations from the 5-contrast multi-atlas approach (FA, MD, vector elements x, y, and z, combined), with those obtained from the same multi-atlas but with only a single contrast — FA-only, MD-only, and a three-contrast approach — EV-only (x, y, z combined). These four methods vary from each other only in the computation of Eq. (14). To compare the four methods statistically, for each structure, we performed a one-way ANOVA to examine significant difference among the Dice results obtained from the four approaches. For statistical correlation analysis, we used William's modification of Hotelling's test [72].

For the scan-rescan reproducibility test, we investigated the volume difference between the automated parcellations of the same structure from the two scans for the same subject. The volume difference is computed as: 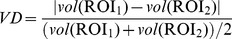, where 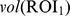
denotes the volume of a specific ROI for scan 1 and 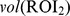
denotes the volume of the same ROI for the second scan of the same subject. In addition, we examined the difference between the mean FA value of the automated parcellation of each single structure for the first scan and that of the automated parcellation for the second scan, as well as the difference between the mean MD values.

## Results

**Figure 1:**
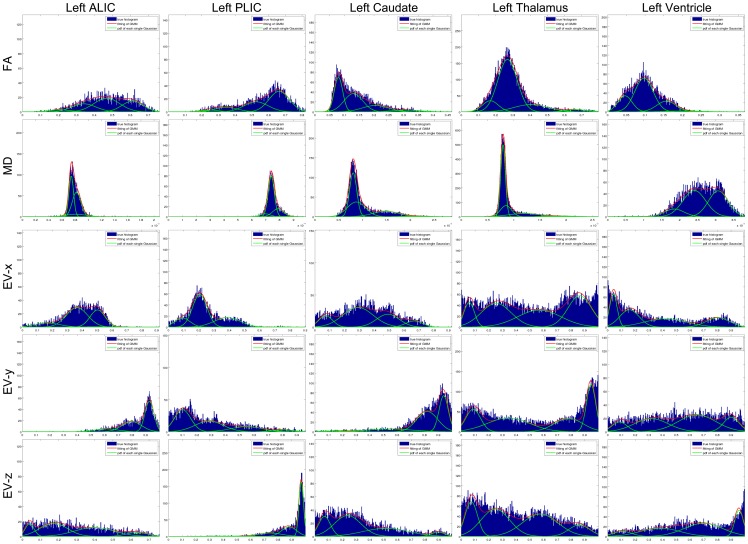
Demonstration of the unique anatomical features revealed by multi-contrast images generated in DTI and GMM. Histograms of the five contrasts, FA, MD, EV-x, EV-y, and EV-z, of five adjacent structures are shown, including two white matter structures (the ALIC and the PLIC), two gray matter structures (the caudate and the thalamus), and the ventricle. In each subplot, blue indicates the histogram of the corresponding contrast within that specific structure, green represents the probability density of each single Gaussian, and red shows the weighted sum of all Gaussians. Abbreviations are: ALIC: Anterior limb of internal capsule and PLIC: Posterior limb of internal capsule.

Figure 1 demonstrates the concept of the multi-contrast image parcellation using five different contrasts obtained from DTI as well as the concept of characterizing the intensity distribution of each contrast using a GMM. The five selected structures are spatially adjacent to each other and need to be accurately demarcated based on their contrast features. Each of the five contrast rows cannot uniquely differentiate all the five structures, but each column (structure) has a unique contrast signature by combining these five contrasts. For example, the distinction between the tissue and the ventricle is most clear on the MD image, while the distinction between the caudate and the anterior limb of internal capsule (ALIC) is most clear on the FA image. Likewise, the difference between ALIC and the posterior limb of internal capsule (PLIC) is largest in the eigenvector image. The GMM quantitatively characterizes the contrast features delineated by these multi-channel histograms.

**Figure 2:**
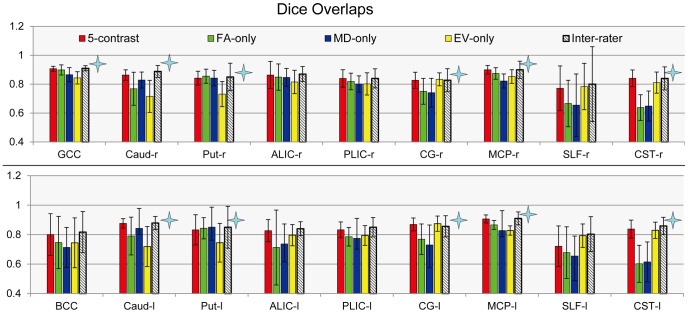
A comparison of parcellating using a single-contrast image and multi-contrast images, in terms of overlap accuracy. The mean Dice overlaps and the standard deviations of the eighteen ROIs obtained from automated parcellations based on five contrasts (red), the single FA contrast (green), the single MD contrast (blue), the vector x y z contrasts (yellow), as well as the inter-rate (patterned). The mean values are calculated across fourteen different subjects. Star marks indicate significant difference among the four sets of Dice results by ANOVA (*p*<<0.05). Abbreviations are: GCC – genu of corpus callosum; BCC – body of corpus callosum; Caud – caudate; Put – putamen; ALIC – anterior limb of internal capsule; PLIC – posterior limb of internal capsule; CG – cingulate gyrus; MCP – middle cerebellar peduncle; SLF – superior longitudinal fasciculus; CST – corticospinal tract.

Figure 2 shows results of Dice measurements, reporting spatial agreement with manual delineation. The 5-contrast approach is compared with FA-only, MD-only, and EV-only approaches. Because of the unique contrast signature of each structure, the best contrast that can accurately define it varies. For example, to define the contricospinal tract (CST), the EV provides the best accuracy, but it provides poor results to define the putamen, which is best defined by FA or MD. The 5-contrast approach performs well for all structures. According to the results from the one-way ANOVA, we found statistical differences among the 4 approaches in 11 structures, in which the 5-contrast approach consistently achieved one of the best results. These structures include: the caudate, the putamen, the cingulate gyrus, the middle cerebellar peduncle, and the corticospinal tract in both hemispheres. The absolute Dice level was 0.8-0.9. Note that some structures are difficult to define even manually with perfect reproducibility. A good example is the superior longitudinal fasciculus (SLF), which has a vague structural boundary and the inter-rater variability is large (Dice = 0.8+/−0.259). Because the manual definition is used as the gold standard, the spatial matching cannot be better than the inter-rater spatial matching (automated methods cannot be more accurate than manual delineation by definition).

**Figure 3:**
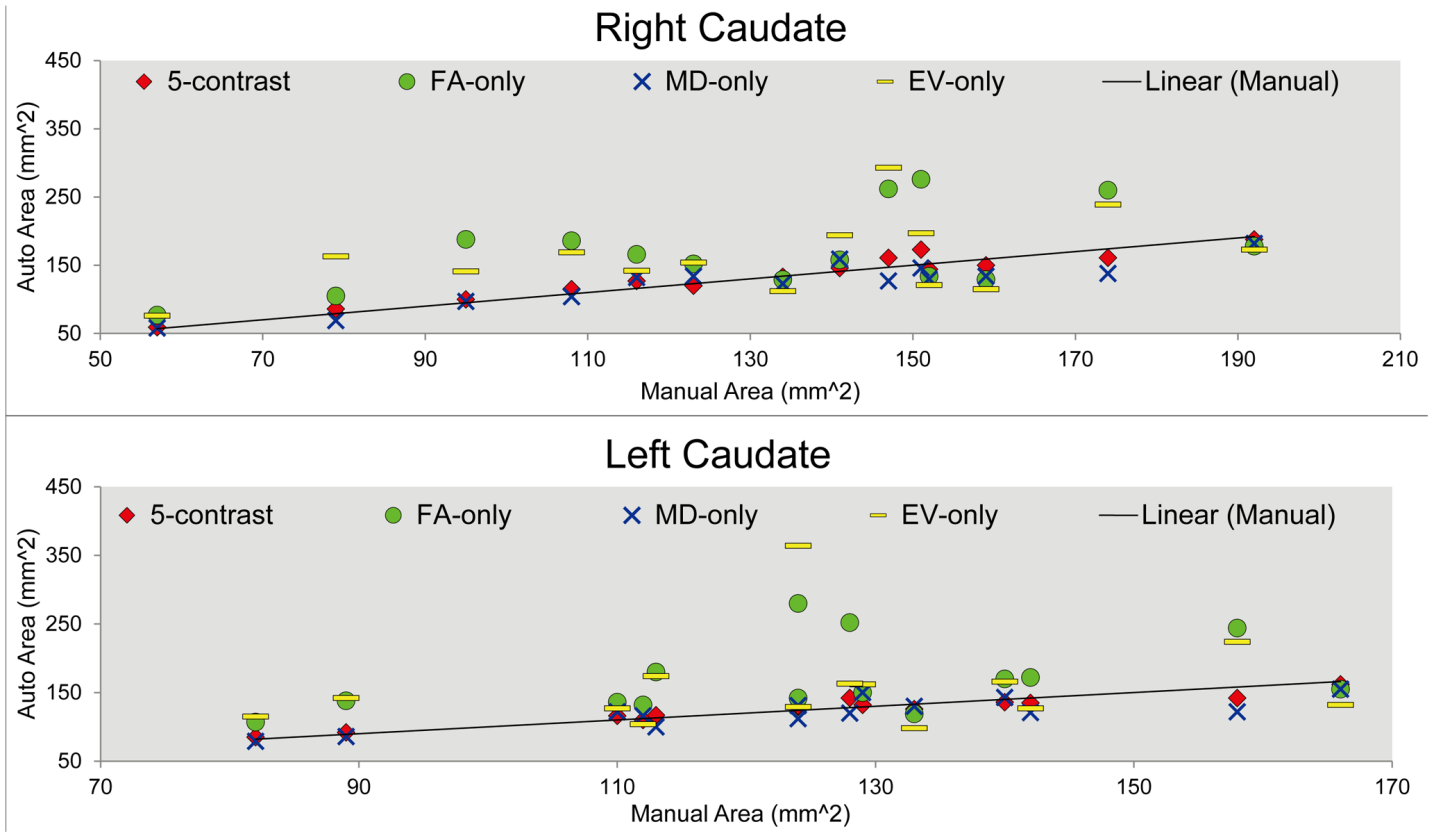
A correlation comparison of the automated caudate parcellation from using a single-contrast image and multi-contrast images. A plot of the correlation between the automated and the manual measurements of the size of the caudate in both hemispheres in square millimeters. Results from the four automated parcellation methods are compared: 5-contrast (red), FA-only (green), MD-only (blue), and EV-only (yellow).

**Figure 4:**
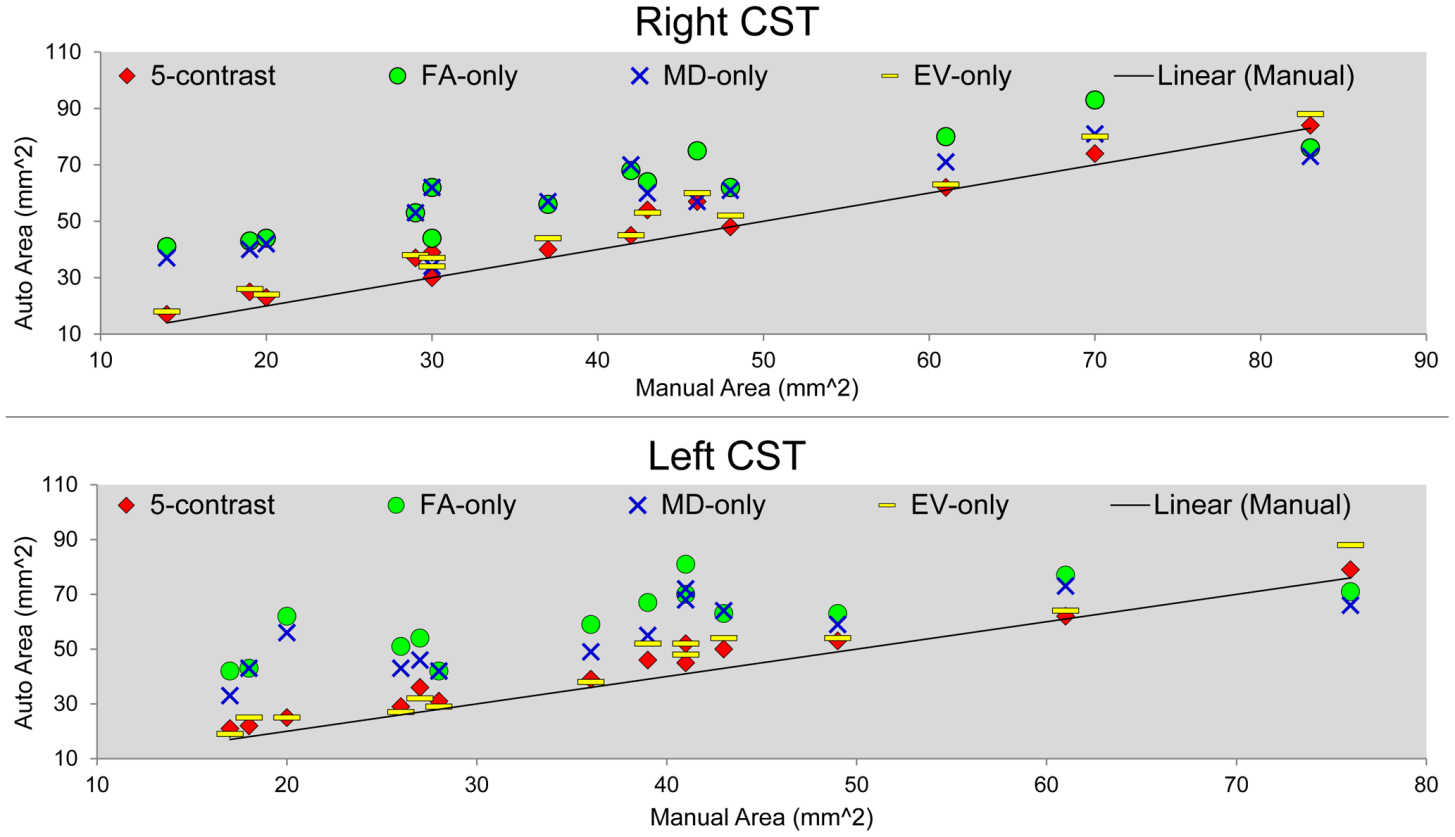
A comparison of the CST correlation obtained from using a single-contrast image and multi-contrast images. A correlation plot between the automated and manual measurements of the sizes of left and right corticospinal tracts (CST). Results from the four automated parcellation methods are compared: 5-contrast (red), FA-only (green), MD-only (blue), and EV-only (yellow).

The correlation coefficients between the sizes of the manual and the automated parcellations obtained from the four approaches for all the eighteen ROIs are listed in Table 3. For some structures, the ROI sizes from all the four automated approaches are highly correlated with the ROI sizes of the manual delineations. However, structures such as the caudate, the corticospinal tract (CST), and the cingulate gyrus (CG), the performance varies from approach to approach. In Figure 3 and Figure 4, we show examples of the correlation plot between the automated and manual approaches for a gray matter (caudate) and a white matter (CST) structures.

**Table 3:** Pearson correlations between manual and four different automated ROI area measures, with bold typesetting indicating that the correlation between the automated and the manual measures is statistically significantly stronger than other methods (*p<0.025*).

	GCC	Caud-r	Put-r	ALIC-r	PLIC-r	CG-r	MCP-r	SLF-r	CST-r
5-contrast	**0.79541**	**0.96321**	**0.94928**	0.98448	**0.95195**	**0.83631**	0.89982	**0.89484**	**0.94142**
MD-only	0.62269	0.91332	**0.93717**	0.98027	0.89410	0.09248	0.82841	0.59750	0.76529
FA-only	**0.78993**	0.55538	**0.95956**	0.97514	0.90322	0.21467	0.87219	0.69858	0.76673
EV-only	0.69702	0.49411	0.90325	0.96372	0.88237	**0.87709**	0.88976	**0.94912**	**0.94356**

	BCC	Caud-l	Put-l	ALIC-l	PLIC-l	CG-l	MCP-l	SLF-l	CST-l
5-contrast	**0.89594**	**0.93600**	**0.95744**	0.95239	**0.88133**	**0.87250**	**0.73939**	**0.82013**	**0.97244**
MD-only	0.76266	0.87585	**0.95652**	0.97787	0.82933	0.42429	0.32009	0.66408	0.71542
FA-only	**0.91011**	0.24694	**0.92485**	0.94455	**0.89486**	0.79367	0.56765	0.61415	0.69422
EV-only	0.75028	0.02319	0.84685	0.96548	0.79192	**0.85428**	**0.78414**	**0.86526**	**0.96540**

**Figure 5:**
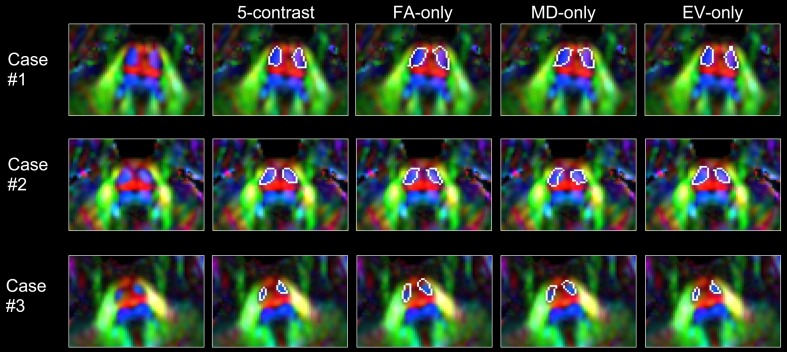
Examples of CST parcellations from single- and multi-contrast approaches. Demonstration of the parcellation accuracy of the CST in three representative cases with different degrees of anatomical abnormalities. Results from five different approaches are compared.

Figure 5 shows actual parcellation results of the CST in the brainstem of subjects with different degrees of abnormalities, which demonstrates how the integration of five contrast information can accurately delineate the sizes. In this example, the fiber-orientation information in the EV contrast is necessary to accurately reflect the small CST sizes in Case #3. Namely, the CST has a characteristic Z-orientation (blue) fiber orientation, which can uniquely differentiate the CST from the surrounding high-FA white matter structures. The integration of the EV information provides strong constraints for the parcellation, specifically defining the high-FA regions with a strong orientation alignment along the Z axis. The FA-, MD-, and EV-only approaches extracted the CST accurately for Case #1 and #2, but grossly overestimated the CST size for Case #3.

Based on the comparison results shown in Table 3, significant improvement of the correlation between the size of the automated parcellations and that of the manual delineations was achieved by the 5-contrast approach, compared with the other single contrast approaches. Likewise, we performed the manual-auto correlation analyses of the mean FA and MD values within each single ROI. As shown in Table 4 and 5, again, the 5-contrast approach is consistently superior to the other three approaches in terms of either FA or MD correlation.

**Table 4:** Pearson correlations between the mean FA value within the manual ROI and the automated parcellations, with bold typesetting suggesting that the correlation between the automated and the manual measures is statistically significantly stronger than other methods (*p<0.025*).

	GCC	Caud-r	Put-r	ALIC-r	PLIC-r	CG-r	MCP-r	SLF-r	CST-r
5-contrast	0.96353	**0.86136**	0.86526	0.98531	0.82955	**0.88852**	**0.94208**	**0.98440**	0.99331
MD-only	0.92838	**0.86687**	0.88287	0.98203	0.76854	0.78890	0.78991	0.83956	0.97452
FA-only	0.97415	**0.91417**	0.89183	0.99429	0.81501	**0.89213**	**0.90952**	**0.98081**	0.97258
EV-only	0.91020	0.81890	0.82255	0.98156	0.78727	**0.90313**	**0.89046**	**0.98859**	0.99241

	BCC	Caud-l	Put-l	ALIC-l	PLIC-l	CG-l	MCP-l	SLF-l	CST-l
5-contrast	**0.98069**	**0.93178**	**0.92879**	**0.98209**	**0.92993**	**0.96545**	**0.93396**	**0.95663**	0.99482
MD-only	**0.99483**	0.83253	**0.93611**	0.93222	0.80897	0.76491	0.81619	0.89348	0.98175
FA-only	0.89738	**0.94095**	**0.93105**	0.95707	**0.91033**	**0.97549**	**0.92899**	**0.95213**	0.96036
EV-only	**0.99254**	0.77616	0.82936	**0.97716**	**0.95108**	**0.94859**	**0.89735**	**0.95659**	0.99355

**Table 5:** Pearson correlations between the mean diffusivity (MD) value within the manual ROI and the automated parcellations, with bold typesetting indicating that the correlation between the automated and the manual measures is statistically significantly stronger other methods (*p<0.025*).

	GCC	Caud-r	Put-r	ALIC-r	PLIC-r	CG-r	MCP-r	SLF-r	CST-r
5-contrast	**0.97192**	**0.94152**	0.99307	0.99115	0.98541	0.93410	**0.94336**	0.98455	**0.98557**
MD-only	0.94154	**0.88121**	0.99237	0.99452	0.97074	0.89304	0.81370	0.95021	0.89566
FA-only	0.95861	0.58329	0.99361	0.99191	0.97357	0.92795	0.90926	0.98013	0.76053
EV-only	0.88039	0.58453	0.98699	0.97750	0.97737	0.92538	0.84391	0.98684	0.91718

	BCC	Caud-l	Put-l	ALIC-l	PLIC-l	CG-l	MCP-l	SLF-l	CST-l
5-contrast	0.94524	**0.95291**	0.99699	**0.98404**	0.98605	**0.98599**	0.92627	0.95778	**0.97023**
MD-only	0.93038	**0.92279**	0.99173	**0.98246**	0.97558	**0.97598**	0.88370	0.95810	0.89994
FA-only	0.98557	0.81496	0.99083	0.10983	0.96575	0.93071	0.83919	0.96408	0.62843
EV-only	0.92346	0.87005	0.99003	**0.96607**	0.97322	0.73985	0.90548	0.98875	0.87714

**Figure 6:**
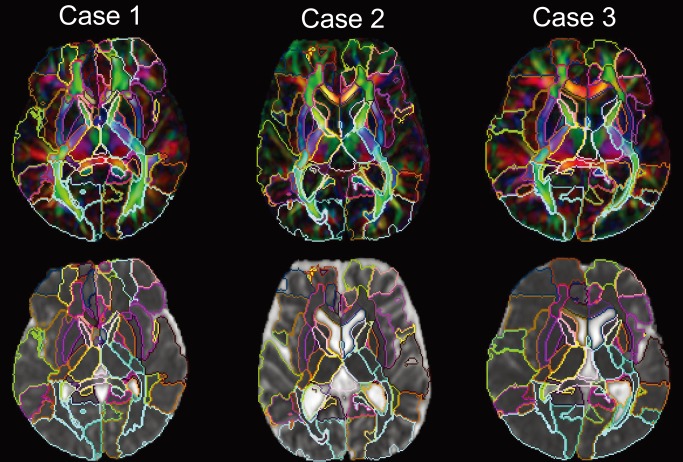
Examples of whole brain parcellations. Results of the whole brain parcellations into 159 structures in three representative cases with large anatomical variability. The parcellation results are superimposed on color (upper row) and MD (bottom row) images.

Figure 6 demonstrates parcellation results for three patients with different degrees of abnormalities. A high level of parcellation accuracy is visually appreciable for the wide variety of anatomical states.

According to our test-retest experiments, the reproducibility results were 4.7%, 2.19%, and 2% for the volume, FA, and MD, respectively, averaged over all 193 structures. If we remove 26 small gray matter structures (<1000 mm^3^), the reproducibility improves to 3.73%, 1.91%, and 1.79% respectively. These small gray matter structures had poor test-retest reproducibility because they lack clear contrasts in DTI and they are usually not the targets of DTI measurements.

**Figure 7:**
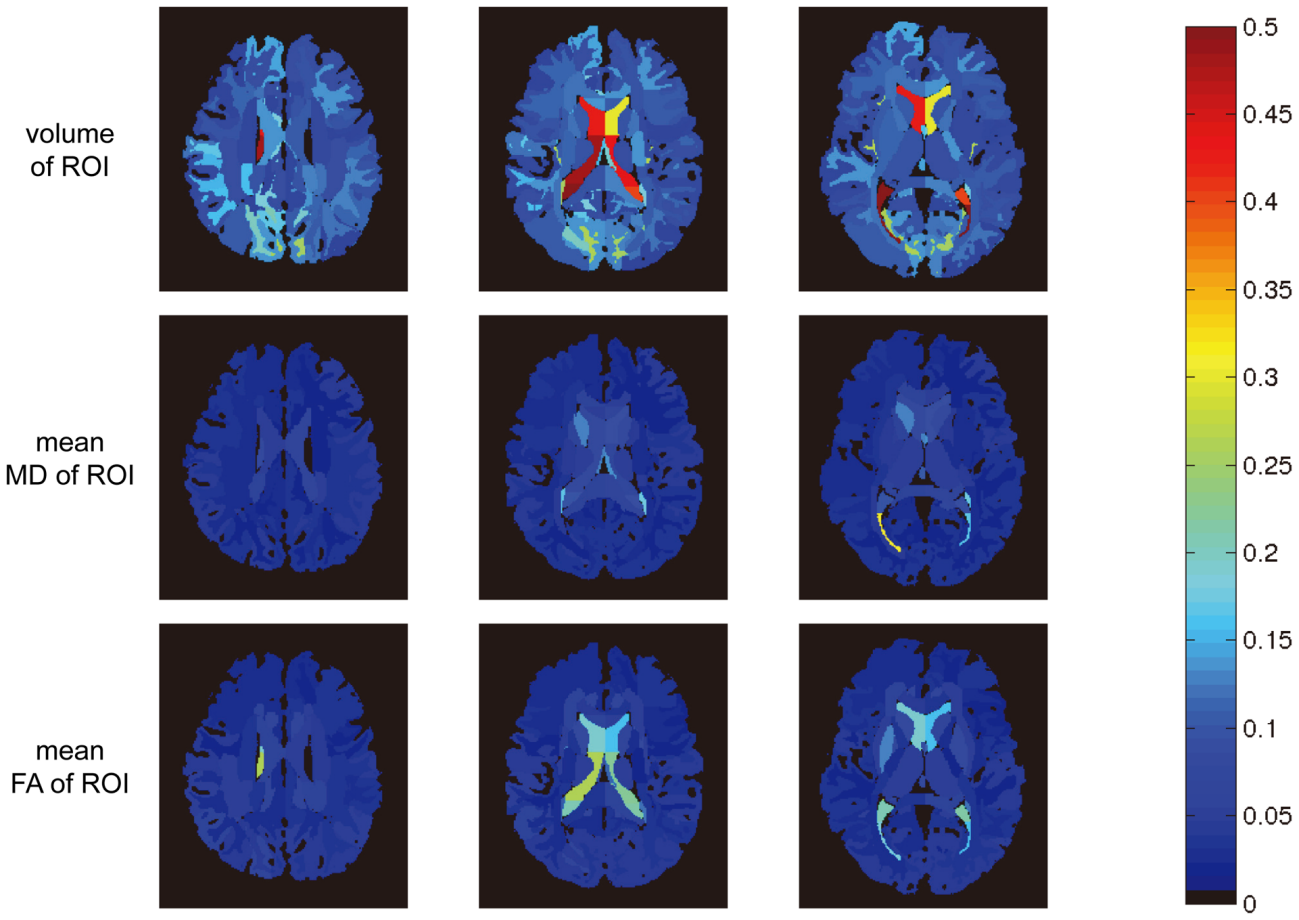
Depiction of the cross-subject variability in different whole brain structures of interest. Demonstration of the cross-subject variability (std/mean) within the 16 healthy subjects for each of the 193 anatomical regions. The population variability in terms of ROI volume (top row), the mean MD of each ROI (middle row), and the mean FA of each ROI (bottom row) are shown. The results are presented for three difference axial slices using a colormap with the color scale ranging from 0 to 0.5.

Figure 7 shows the maps of cross-subject variability in the volume, FA, and MD measurements. The cross-subject variability is computed for each label of interest. It is quantified as the ratio of the standard deviation to the mean value across the sixteen subjects. A large amount of morphological variability was found in the ventricle volumes, while the standard deviations of the volumes of white matter structures are in 10-20% range. The standard deviations of the FA and MD were noticeably lower and most areas were below 10%. The table in Appendix shows a comprehensive report of the test-retest reproducibility and the cross-subject variability of all the 193 defined structures. These values should provide useful information for power calculation in future study designs.

## Discussion

In this study, we developed and tested an automated image parcellation method based on a multi-contrast multi-atlas likelihood-fusion algorithm. DTI can generate multiple quantitative maps with markedly different qualities of anatomical contrasts. The mean diffusivity contrast provides clear distinction between the tissue (generally within the range of 0.6–0.9 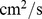) and the CSF (approximately 3.0–3.5 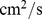), providing a strong constraints to define the ventricles and the brain surface. The FA contrast provides sharp distinction between the gray (typically FA<0.15–0.25) and white matter structures (FA>0.15–0.25). The eigenvector (EV) can differentiate intra-white matter structures based on their characteristic orientations. In this work, we used the absolute values of the three components, EV-x, EV-y, and EV-z. While this approach solves the difficulties associated with the sign of the eigenvectors, some orientation information degenerates [73]. This is obviously a simplified approach and there is room for improvement. The mixture of these three types of information could also invite noise. For example, in the low-FA gray matter structures, the fiber orientation information may be random and should not receive significant weighting. This type of weighting is naturally achieved by incorporating the variability information about the voxel values within a single parcellated structure; for example, the EV information of the thalamus in Figure 1 shows almost equal values for the X, Y, and Z channels with high intra-structure variability. In Eq. (18), we use mixtures of Gaussians to model the intensity distribution within a single structure. If the intensity within the structure is homogeneous, the algorithm automatically assigns weight 1 to a single Gaussian. If there is high intra-structure variability, multiple Gaussians will be used to model the intensity, with each Gaussian being given a small weight. In this way, it effectively reduces the contribution of this contrast information in computing the quantity 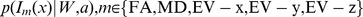 in Eq. (14). By incorporating the consistent anatomical signatures into the parcellation criteria, we aim to achieve robust parcellation. In the past, multi-contrast image registration approaches have been postulated including ones for DTI data [60], [74]–[79]. These tensor- or vector-based registration also indicated improved registration accuracy [56]–[59]. The proposed method can be considered as an extension of these previous works by incorporating them into a multi-atlas framework.

The improved accuracy, with respect to a single-contrast approach, is shown in Figure 2 using Dice measurements. While the performance of single-contrast approaches varies depending on the structure, the five-contrast approach consistently achieved the highest level of accuracy. As mentioned in the Results section, the accuracy of the automated method cannot be higher than the reproducibility level of the manual delineation. In this sense, the results in Figure 2 indicate that the five-channel approach is as good as a human rater. Careful observation of this figure reveals that, for many core white matter structures with distinctive and uniform tract orientations, the eigenvector contrast alone can provide a similar level of accuracy as the five-contrast method, suggesting that the parcellation was mainly driven by the eigenvector contrast. However, if a structure is not characterized by a uniform fiber orientation due to curvature within a segment, such as GCC and BCC, the eigenvector may not always be the most reliable contrast.

While the Dice measurements (Figure 2) provide important information about the accuracy level of the automated parcellation, it is probable that the correlation results reported in Table 3 are more important for actual image-based studies. Anatomical delineations of brain structures depend on anatomical definitions. It is reasonable that there is consistent difference in boundaries of defined structures between two different approaches. In this sense, low Dice values do not necessarily mean that the automated results are not useful. The high correlation between the manual and automated methods indicates that they have similar powers to differentiate different anatomical states, which is ultimately the goal of quantitative analyses. In this respect, the high correlation between the 5-contrast and manual approaches is an encouraging result.

One limitation of our accuracy evaluation is that the measured structures were limited to the core brain structures, which can be reproducibly defined manually. This is inevitable because the manual delineation results were used as the gold standard. As reported in the Results section, one of the core white matter structures, the SLF, suffered from low inter-rater reproducibility due to its complex shape. Reproducible definitions of peripheral white matter regions by manual tracing would be prohibitively difficult and, due to the absence of the gold standard, the accuracy measurements of the proposed automated segmentation were challenging. In this study, we are therefore limited to reporting test-retest reproducibility and population variability measurements, which could be important resources for power analyses of the proposed method.

The test-retest reproducibility showed less than 5% variability for the volume measurement for most of the defined structures (see Appendix table).The test-retest reproducibility measures of FA and MD indicated higher reproducibility (less than 3%). The anatomical variability for the 193 measured structures reported in Figure 7 should provide information for power analysis to design population studies.

In this study, we reported the accuracy level of the multi-contrast multi-atlas approach for a wide range of anatomical phenotypes. The performance of this technology relies heavily on the availability of atlases with consistent parcellation criteria. Creation of such atlases is a time consuming task, which is one of the limitations of this approach. The accuracy of the parcellation is, of course, influenced by the extent of the anatomical abnormality. Conceptually, the greater the number of the atlases and the wider the anatomical range the inventory includes, the wider the applicability of the tools regardless of the anatomical findings/pathology. However, the larger number of the atlases would cost computational time. In the current study, we used sixteen atlases. The relationship between the number and properties of the atlases and the resultant accuracy and applicable anatomical range is not systematically analyzed in this study, which is an important future investigation.

An interesting extension of this discussion is the necessity of a “normal” definition. Usually, when we establish a population-based atlas, we invest great efforts to make sure the population consists of real, normal, healthy subjects. The involvement of abnormal cases in the atlas building would make the atlas biased. In the proposed multiple-atlas approach, we need to make sure that the atlases cover a wide range of anatomical phenotypes, at least if the approach is to have clinical applications; if a structure of interest is dislocated to a position beyond the range of all atlases, the likelihood would become zero and we cannot get correct parcellation. In this process, the achievement of accurate labeling is an independent issue from whether we define the normal anatomy in the atlases.

In conclusion, we developed a multi-contrast multi-atlas image parcellation algorithm and applied it to whole brain parcellations in DTI data. Compared to single-contrast approaches, improved parcellation accuracy was confirmed. Anatomical structures in patients with a wide range of anatomical states could be accurately parcellated by incorporating various anatomical phenotypes in the atlas inventory.

## Supporting Information

Appendix S1 **Quantification of the test-retest reproducibility and the cross-subject variability in the volumetric measurement, the mean FA value, and the mean MD value, for each of the 193 ROIs.**
(XLSX)
